# Radiation Effects of Normal B-Lymphoblastoid Cells after Exposing Them to Low-Dose-Rate Irradiation from Tritium β-rays

**DOI:** 10.3390/biology13060418

**Published:** 2024-06-05

**Authors:** Bing Deng, Yi Quan, Zhilin Chen, Heyi Wang

**Affiliations:** Institute of Nuclear Physics and Chemistry, China Academy of Engineering Physics, Mianyang 621900, China; quanyi-2008@126.com (Y.Q.); chenzhilin@caep.cn (Z.C.); hywang@caep.cn (H.W.)

**Keywords:** tritium, low dose, radiation effect, B-lymphoblastoid cell

## Abstract

**Simple Summary:**

Tritium is one of the most significant fuels in fusion reactors and it also represents an important radioactive isotope discharged into the environment from nuclear power plants. The risk of tritium should be answered before the construction of fusion reactors. Mitochondria have been identified as a potentially sensitive target for investigating low-dose/low-dose-rate radiation effects, with extensive experimental results obtained using X-ray irradiation. In this manuscript, we experimentally studied the impact of mitochondrial function regulation on tritium radiation biological effects. It was found that there was no significant difference in cell viability induced by different doses. However, the results of ATP levels showed a considerable difference after irradiation at a dose of 500 mGy by tritium β-rays compared to the sham-irradiated sample, while the levels obtained with X-ray irradiation were almost identical to the sham-irradiated sample. In contrast, ATP levels for both tritium β-rays and X-rays at a dose of 1.0 Gy showed little difference compared to the sham-irradiated sample. This suggests that mitochondria might be a potentially sensitive target for investigating tritium β-ray irradiation effects, and they are helpful for the risk evaluation of tritium for the development of fusion energy.

**Abstract:**

The effects of tritium at low doses and low dose rates have received increasing attention due to recent developments in fusion energy and the associated risks of tritium releases into the environment. Mitochondria have been identified as a potential candidate for studying the effects of low-dose/low-dose-rate radiation, with extensive experimental results obtained using X-ray irradiation. In this study, irradiation experiments were conducted on normal B-lymphoblastoid cells using HTO at varying doses. When compared to X-ray irradiation, no significant differences in cell viability induced by different doses were observed. However, the results of ATP levels showed a significant difference between the irradiated sample at a dose of 500 mGy by tritium beta-rays and the sham-irradiated sample, while the levels obtained with X-ray irradiation were almost identical to the sham-irradiated sample. In contrast, ATP levels for both tritium beta-rays and X-rays at a dose of 1.0 Gy showed minimal differences compared to the sham-irradiated sample. Furthermore, distinct effects at 500 mGy were also confirmed in both ROS levels and apoptosis results obtained through tritium beta-ray irradiation. This suggests that mitochondria might be a potential sensitive target for investigating the effects of tritium beta-ray irradiation.

## 1. Introduction

Tritium is one of the most significant fuels in fusion reactors, and it is also an essential radioactive isotope discharged into the environment from nuclear power plants, particularly for CANDU-type reactors. Due to growing concerns about the impact of tritium on the environment and human health, tritium’s risk has garnered increased attention from academic researchers and the public [1,2]. A multitude of experiments have suggested that the radiation hazard of tritium might be underestimated, and its relative biological effectiveness (RBE) for tritium β-rays should lie between 1.0 and 5.0, rather than the 1.0 specified in the ICRP 103 report [3,4,5]. In most cases, tritium enters the human body in the form of tritiated water (HTO). Although HTO has a rapid biological half-life, organically bound tritium (OBT) produced in the human body as a result of exchanges with hydrogen in biological molecules exhibits long-term biokinetics and has the potential to cause persistent low-dose rate radiation [6,7]. Therefore, it is crucial to focus on the prolonged low-dose effects induced by tritium β-rays compared to other acute irradiations. To date, numerous tritium irradiation experiments have been conducted using mice, including investigations into the radiation damage effects on corresponding cells in peripheral blood [8], the nervous system [9], the immune system [10], the lymphatic system [11], and the reproductive system [12,13], to enhance our understanding of the teratogenic, carcinogenic, genetic, and reproductive effects of tritium irradiation [14,15]. However, some uncertainty persists regarding the effects at low doses/low-dose rates, alike for tritium exposure. For instance, Guéguen et al. reviewed in vivo animal studies on low levels of HTO conducted within an international collaboration, and their results indicated that some cellular and molecular responses to tritium seemed to be beneficial, while others were potentially detrimental [2]. Currently, no conclusive answer can be provided regarding whether low dose/low-dose rate radiation from tritium exposure is harmful or beneficial to human health. Consequently, ongoing research is required to explore the potential mechanisms regulating the occurrence of low-dose effects from tritium β-ray radiation in order to shed light on these uncertainties.

To better understand the hazard of tritium, experiments with high-sensitivity systems, such as those used to probe changes in mutation load or frequency, are conducted to amplify the biological effects induced by low-dose/low-dose-rate radiation. Tauchi transfected the Hprt1 gene [16], which is responsible for encoding the human X chromosome into Hprt1-defective hamster cells through gene shear and other methods, thereby creating a mutation-prone experimental system (sensitivity increased by approximately 50 times). Umata found that the background mutant frequency induced by HTO exposure in a p53-deficient system was 1.7 times larger than that exposed to chronic 137Cs γ-rays [17]. These hypersensitive assay systems suggest that using defective systems to study low-dose effects induced by HTO exposure may help uncover more key events involved in the regulatory mechanism. It also benefits our understanding of why tritium exposure has higher relative biological effectiveness values at low doses, particularly when compared to X/γ-rays. Furthermore, with the increasing knowledge of non-targeted effects induced by low-dose radiation, it has been demonstrated that, in addition to the cellular nucleus, mitochondria are another crucial component associated with damage signal transmission [18,19]. Kulkarni et al. found that mitochondrial mutant cells with mutations occurring at different mitochondrial DNA (mtDNA) sites exhibited distinct radio sensitivities to X-ray irradiation, which was related to diverse DNA damage responses triggered in the mitochondrial mutant cells [20]. Currently, many studies focus on nuclear DNA damage caused by tritium exposure [21,22,23], but the impact of mitochondrial function regulation on the biological effects of tritium radiation has not been reported.

In this study, considering the lymphopoietic system’s sensitivity to radiation, including low doses and low-dose rate radiation, irradiation experiments with HTO were conducted on normal B-lymphoblastoid cells. Additionally, an analysis of several biological endpoint responses related to mitochondrial function was performed. Furthermore, the results regarding cell viability and ATP levels after irradiation with HTO were compared with those obtained from X-ray irradiation experiments conducted at the same dose in the reference.

## 2. Materials and Methods

### 2.1. Cell Lines

The normal B-lymphoblastoid cell line GM15036 was obtained from the Coriell Cell Repository. All cells were grown in suspension in RPMI 1640 medium (GIBCO, Paisley, UK), supplemented with 15% fetal bovine serum (GIBCO, Paisley, UK), and 2 mM l-glutamine (GIBCO, Paisley, UK). Cells were sub-cultured by seeding at a concentration of 1.0 × 10^5^ cells/mL and were grown in a fully humidified incubator with 5% CO_2_ at 37 °C in T25 culture flasks (Corning, New York, NY, USA).

### 2.2. Irradiation Protocol

**Figure 1:**
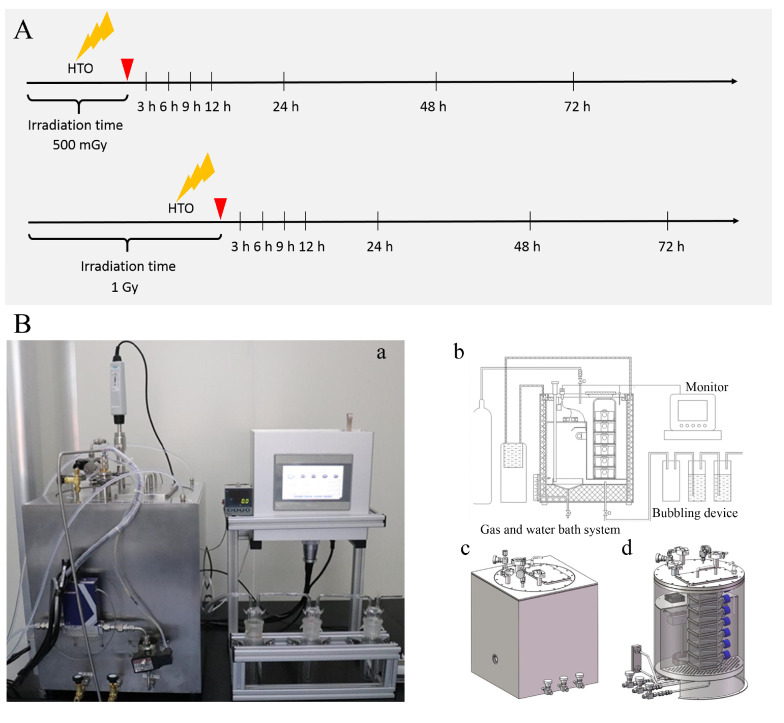
A schematic diagram illustrates the experimental protocol and device for HTO exposure. (**A**) Experimental protocol for HTO exposure. At 0 h, cells in suspension were counted and seeded into T-25 flasks. For HTO-irradiated groups, cells were cultured using the HTO-contaminated medium in the device specifically developed for HTO exposure. When the dose reached 500 mGy or 1 Gy, cells were harvested and rinsed with HTO-free medium twice. Subsequently, the irradiated cells were cultured with HTO-free medium in the device. At each time point, cells were harvested for various assays. Sham-irradiated groups were cultured in a conventional incubator. (**B**) The device developed for HTO exposure: (**a**) a photograph of the device used to culture cells exposed to HTO; (**b**) a schematic diagram illustrating how the equipment functions for incubating cells with HTO; (**c**) a sketch map of the outer shell of the cultivation equipment; (**d**) a sketch map of the internal view of the incubation equipment.

Due to the easy diffusion characteristic of HTO, specific equipment was designed for HTO exposure experiments (as shown in Figure 1). Suspension cells were cultured in T-25 flasks containing 3 mL of medium supplemented with 300 μL HTO of 8.29 × 10^7^ Bq/mL (8.29 × 10^6^ Bq/mL HTO, prepared in our laboratory) for varying time periods (approximately 18.37 h and 36.74 h) to achieve different doses (corresponding to 500 mGy and 1.0 Gy, respectively). Before opening the door to harvest cells from the T-25 flasks, 5% CO_2_ gas was filled in the culture box to remove HTO vapor produced during cell incubation in HTO-contaminated medium. The flushing gas was washed through three bubblers one by one to collect HTO before discharge. Subsequently, cells were transferred to centrifugal tubes and centrifuged at 2000 rpm for 5 min to remove HTO-contaminated medium. They were then rinsed with 10 mL of HTO-free medium twice to eliminate residual HTO. The removal of HTO from cell cultures was monitored by measuring the radioactivity of the rinse medium using a liquid scintillation analyzer (PerkinElmer, Waltham, MA, USA, Tri-Carb 3100TR). The sham-irradiated group underwent the same procedure as the 1 Gy dose for HTO exposure, except that cells were cultured in HTO-free medium and incubated in a standard incubator. The time point when cells were transferred into T-25 flasks to prepare for the HTO exposure experiment was defined as 0 h.

For X-ray irradiation experiments, we referred to the study conducted by Kulkarni et al., using the same type of cell and identical dose. In brief, a 160 kVp Faxitron X-ray unit was employed, delivering 160 kV X-rays at a dose rate of 0.69 Gy/min, resulting in a total of 500 mGy or 1 Gy, as described by Kulkarni et al. [20].

### 2.3. Cell Viability Assay

**Figure 2:**
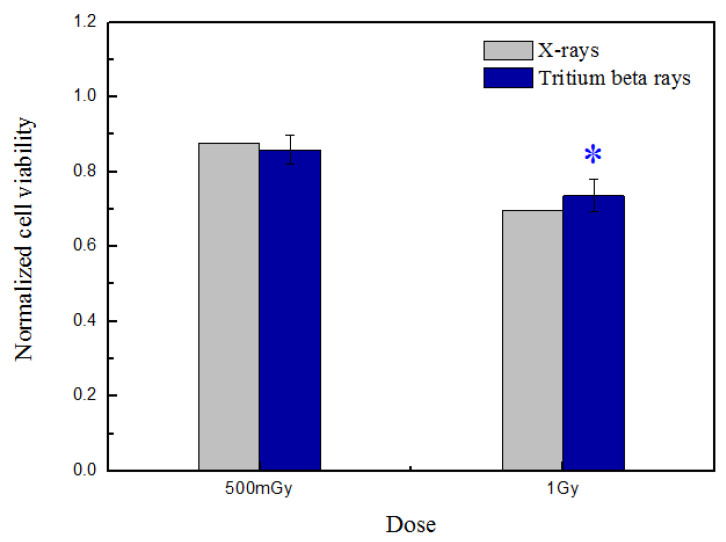
Normalized cell viability was measured at 4 days post-tritium β-ray and X-ray irradiation. After HTO exposure, the normal B-lymphoblastoid cells in T-25 flasks were rinsed twice and resuspended in HTO-free medium. One thousand cells were reseeded per well, with five replicates in 96-well flat-bottom plates, using 200 μL of medium prepared for performing the CCK-8 assay. The plate was scanned at 450 nm, and the obtained data were normalized against the sham-irradiated group. The data are presented as means ± SDs. ‘*’ represented *p* < 0.05, which was considered to be statistically significant.

After HTO irradiation, cells were counted, and 10,000 cells were reseeded per well in five replicates on 96-well flat-bottom microtiter cell culture plates (Nunc). This was followed by 4 days of culture in 200 μL of complete culture medium without HTO. Subsequently, after a 4-day incubation at 37 °C, cell proliferation was determined using a cell counting kit-8 (CCK-8) (Beyotime, Guangzhou, China). In brief, three control wells with HTO-free medium were set up without cells. A total of 10 μL of reagent was added to each well and incubated for 4 h. Then, the plate was scanned using a microplate reader (Spark, Tecan, Mannedorf, Switzerland) at 450 nm. The data presented in Figure 2 were normalized against the sham-irradiated group.

### 2.4. Intracellular ATP Measurements

Intracellular ATP was measured using the CellTiter-Glo ^®^ Luminescent Cell Viability Assay (Promega Inc., Madison, WI, USA) as previously described [20]. After HTO exposure, the cells in a T-25 flask were rinsed twice and resuspended at a density of 2 × 10^5^ cells/mL. Hundred microliters of cells were transferred into a 96-well black plate with five replicates and incubated at the measurement time points (3 h, 6 h, 9 h, 12 h, and 24 h, respectively). The “0 h” refers to the sample being detected immediately after the cells were transferred into the 96-well plate, which is the same as mentioned in the following paragraph. A control well was set up using HTO-free medium without cells to obtain the background luminescence. After the plate was allowed to equilibrate for approximately 30 min at room temperature, 100 μL of CellTiter-Glo ^®^ reagent was added to each well, including the control well. The plate was mixed on an orbital shaker for 2 min to ensure complete cell lysis. Then, the plate was incubated for 10 min at room temperature to stabilize the luminescence signal. The luminescence signal was measured using a microplate reader (Spark, Tecan, Mannedorf, Switzerland) with an integration time of 50 milliseconds per well. The data measured at different time points post-HTO exposure were normalized with the sham-irradiated group detected at 0 h.

### 2.5. Reactive Oxygen Species (ROS) Measurements

Intracellular ROS, represented by its oxidation derivatives, was assessed using 2′, 7′-dichlorodihydrofluorescein diacetates (DCFH) (Beyotime, Shanghai, China). The protocol was adapted from the work of McArdle et al. [24]. In brief, after HTO exposure, cells in T-25 flasks were rinsed twice and incubated in HTO-free medium until harvest. At each measurement time point (3 h, 12 h, 24 h, 48 h, and 72 h, respectively), cells were centrifuged and resuspended in medium supplemented with 10 μM DCFH-DA reagent at a density of 2 × 10^5^ cells/mL. Following this, cells were cultured for 30 min at 37 ℃ and rinsed using fetal bovine serum supplemented medium three times to remove the residual fluorescent probe. Finally, 100 μL of cells were transferred into a 96-well black plate with five replicates. The fluorescence was scanned at 485/520 nm using a microplate reader (Spark, Tecan, Mannedorf, Switzerland).

### 2.6. Apoptosis

Apoptosis was assessed using the caspase-3/7 activity apoptosis assay kit (Sangon Biotech, Shanghai, China). After HTO exposure, the cells in a T-25 flask were rinsed twice and resuspended in HTO-free medium at a density of 1 × 10^5^ cells/mL. Hundred microliters of the cells were transferred into a 96-well poly-D lysine plate, which had five replicates. At each measurement time point (3 h, 9 h, 24 h, 48 h, and 72 h, respectively), 100 μL of a caspase-3/7 assay loading solution was added to each well, including the control well, and the plate was incubated at room temperature for 2 h with light protection. Next, the plate was centrifuged at 800 rpm for 2 min. The fluorescence intensity was monitored using a microplate reader (Spark, Tecan, Mannedorf, Switzerland) at 490/525 nm.

### 2.7. Statistical Analysis

Statistical analyses were conducted on the mean data obtained from a minimum of three independent experiments. The data were presented as the means ± SDs. To detect statistical significance between X-ray radiation and HTO exposure at the same dose in the cell viability assay, a one-way ANOVA test was employed. For comparing the time course of ATP, ROS, and caspase-3/7 activity, the data from different measurement time points or doses were analyzed using a two-way ANOVA test with post-hoc contrasts via OriginPro 8.1 software. ‘*’ represented *p* < 0.05, which was considered to be statistically significant.

### 2.8. Dose Calculations

Dose exposure (D, Gy) in the experiments can be calculated using the following formula:

(1) $$D=\int_{0}^{T}C(t)\bar{E}kdt$$

where *C*(*t*) is the tritium concentration in the tritiated water, Bq/g; $\bar{E}$ is the average energy of beta-rays emitted by tritium, keV; *k* is the transfer coefficient, 1.6 × 10^−13^ J/keV; and *T* is the exposure time, s.

## 3. Results and Discussion

### 3.1. Analysis of Cell Viability Induced by Tritium β-ray Irradiation

Cell viability after tritium β-ray irradiation was measured using the cell proliferation assay, and the results were compared with experiments performed using X-rays at the same dose published in reference [20], as shown in Figure 2.

The results demonstrate a dose-dependent cell-killing trend that intensifies as the dose increases. Nonetheless, there is no significant difference in cell viability between X-rays and tritium beta-rays at the same dose, whether it be 500 mGy or 1 Gy. Considering that the dose rate of tritium β-rays is approximately equivalent to low-dose-rate irradiation, increasing evidence suggests that the mechanism underlying low-dose-radiation effects involves metabolic oxidation reactions [25,26]. Mitochondria are renowned for their role in regulating various metabolic processes, including calcium signaling, redox homeostasis, and cellular apoptosis [27].

Thus, to determine how mitochondria respond to the low-dose-rate radiation produced by tritium β-rays, the influence on mitochondrial function was analyzed after exposing them to tritium β-rays at low and high doses, which was also compared with X-rays.

### 3.2. Mitochondrial Function Related to the Radiation Effects Triggered by the Low-Dose Rate of Tritium β-rays

**Figure 3:**
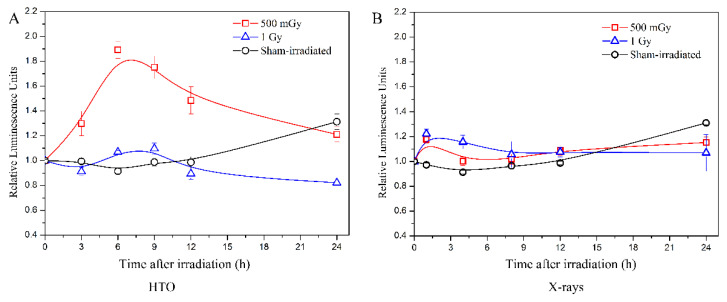
Measurements of intracellular ATP levels induced by tritium β-ray and X-ray irradiation. (**A**) Time course of intracellular ATP levels after HTO exposure. After HTO exposure, cells in T-25 flasks were rinsed twice and resuspended at a density of 2 × 10^5^ cells/mL. Cells (100 μL) were transferred into a 96-well black plate with five replicates and incubated until the measurement time points (3 h, 6 h, 9 h, 12 h, and 24 h, respectively). The ATP levels of cells without any experimental manipulation were detected and presented as the 0 h result. (**B**) Time course of intracellular ATP levels after X-ray radiation. The data refer to the work reported by Kulkarni et al. [20]. All data were normalized with the results at 0 h. Data are presented as the means ± SD. The curves in all figures were obtained from B-spline connection interpolation in Origin 8.1 software.

After HTO exposure, the intracellular ATP levels at the doses of 500 mGy and 1 Gy were examined (Figure 3A), and the results were compared with experiments performed using X-rays at the same dose published in reference [20] (Figure 3B).

The results indicate that after X-ray irradiation at doses of 500 mGy and 1 Gy, there is a slight increase in ATP levels immediately after irradiation. These levels then decrease to those of the sham-irradiated group at 4 h and 8 h, respectively. However, tritium β-ray irradiation at a dose of 500 mGy shows a dramatic increase in ATP levels at 3 h, which lasts until 6 h, followed by a decrease at 9 h. At 24 h post-exposure, the levels return to those of the sham-irradiated group. For the dose of 1 Gy, the trend is similar, but the increase is less pronounced than that at 500 mGy. The increase in ATP levels at 24 h in the sham-irradiated group is possibly due to the increased cell count caused by cellular proliferation. This is because at the beginning, the same number of cells were transferred into five 96-well black plates and cultured in an incubator. At each measurement time point, a 96-well black plate is removed to detect the ATP level. For the 24 h time point, after 24 h of cellular proliferation, the cell quantity is greater than the initial cell number transferred.

Therefore, an elevated ATP level is observed in the sham-irradiated group at each measurement time. Comparing the results between tritium β-rays and X-rays, the ATP level increase at a dose of 500 mGy is significantly higher, and this high ATP level lasts longer in the tritium β-ray group. For a dose of 1 Gy, the time course of ATP levels measured in both tritium β-ray and X-ray groups is similar, but the time when the ATP level begins to increase is earlier in the X-ray group compared to the tritium β-ray group. These results suggest that although there is little difference in cell viability between tritium β-rays and X-rays, the post-treatment ATP levels show dramatic differences at a dose of 500 mGy. Moreover, tritium β-rays exhibit a moderate influence at a high dose of 1 Gy. Compared to X-ray irradiation, the ATP level of B-lymphoblastoid cells after tritium β-ray irradiation shows a different response at the same dose, whether it be 500 mGy or 1 Gy. The time for the ATP level increase is later, and a longer persistence of high ATP levels is observed for tritium β-ray exposure. This implies that there was an acute response to X-ray irradiation, whereas tritium β-rays induced a prolonged effect.

**Figure 4:**
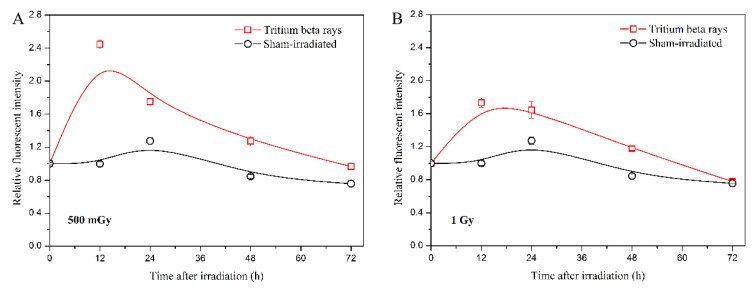
Analysis of intracellular ROS levels induced by tritium β-ray irradiation. (**A**) Time course of ROS levels at a dose of 500 mGy; (**B**) time course of ROS levels at a dose of 1 Gy. After HTO exposure, cells in T-25 flasks were rinsed twice and incubated in HTO-free medium until harvest. At each measurement time point (3 h, 12 h, 24 h, 48 h, and 72 h, respectively), cells were centrifuged and resuspended in medium supplemented with 10 μM DCFH-DA reagent at a density of 2 × 10^5^ cells/mL. After incubating with the fluorescent reagent for 30 min, cells (100 μL) were transferred into 96-well black plates with five replicates, and the fluorescence was scanned at 485/520 nm on a microplate reader. Data are presented as the means ± SDs. The curves in all figures were obtained from B-spline connection interpolation in Origin 8.1 software.

In addition, the ROS level serves as another representative characteristic of mitochondrial function disruption [28,29]. ROS also acts as a messenger, transmitting intercellular signaling with low-dose effects [30,31]. Therefore, detecting the ROS level helped confirm the persistently high level of ATP observed in the HTO exposure group. The results are shown in Figure 4.

The results indicate that the time course of ROS levels at a dose of 500 mGy is similar to the trends observed in the ATP level at the same dose. The ROS levels initially increase significantly and then gradually decrease to the sham-irradiated level (Figure 4A). However, for tritium β-ray exposure, the maximum ROS level at 500 mGy is reached at 12 h, which is later than the maximum ATP level at the same dose. At a dose of 1 Gy, the ROS level starts to decrease 12 h after HTO exposure (Figure 4B), which is later than the decrease in ATP level. The results suggest that the increase in ATP level after HTO exposure triggers an increase in cellular ROS. The early decrease in ROS level compared with sham-irradiation observed might be associated with the low-dose-rate radiation effects. Given that the exposure duration for 1 Gy is approximately twice that of 500 mGy, the DNA damage response is likely initiated during HTO exposure. Consequently, radical scavengers and antioxidants can be activated to protect cells from subsequent events [31].

**Figure 5:**
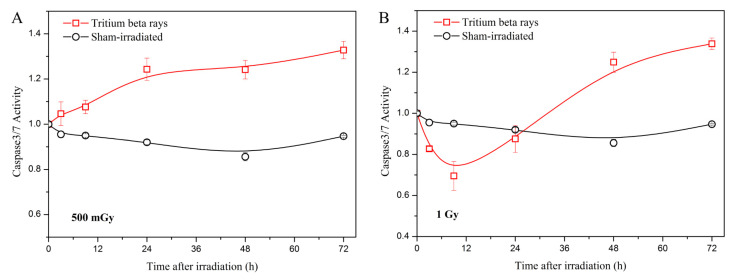
Caspase-3/7 green detection in the normal B-lymphoblastoid cell line GM15036 following exposure to tritium β-rays. (**A**) Time course of caspase-3/7 activity at a dose of 500 mGy; (**B**) time course of caspase-3/7 activity at a dose of 1 Gy. After HTO exposure, the cells in a T-25 flask were rinsed twice and resuspended in HTO-free medium at a density of 1 × 10^5^ cells/mL. One hundred microliters of cells were transferred into a 96-well poly-D lysine plate, which contained five replicates. At the measurement time points (3 h, 9 h, 24 h, 48 h, and 72 h, respectively), the plate was scanned using a microplate reader at 490/525 nm. The curves in all figures were obtained using B-spline connection interpolation in Origin 8.1 software.

Considering that mitochondrial function is involved in cell apoptosis, which is a major form of cell death [32], the analysis of apoptosis was conducted by measuring the activity of caspases 3/7. The results are presented in Figure 5.

The results indicate that at a low dose of 500 mGy, apoptosis increases with time following HTO exposure (Figure 5A). In contrast, at a high dose of 1 Gy, apoptosis decreases at 3 h and increases from 9 h to 72 h post-treatment (Figure 5B). Furthermore, there is no significant difference in apoptosis between the 500 mGy and 1 Gy doses at 72 h post-exposure. Considering the results of ATP and ROS levels, the increased apoptosis after HTO exposure potentially correlates with mitochondrial dysfunction, as mitochondrial outer membrane permeabilization can initiate apoptosis through an intrinsic pathway [33]. Apoptosis has also been shown to be related to mitotic death in irradiated cells [34]. Consequently, a decline in cell viability is observed at both the 500 mGy and 1 Gy doses. Although apoptosis measurements are similar between 500 mGy and 1 Gy at 72 h, cell viability at the 1 Gy dose is lower than that of 500 mGy. Given that cell viability is also regulated by the DNA damage response, and high levels of ROS can cause DNA damage by attacking it, we speculate that this may be one reason why cell viability at the 1 Gy dose is lower than that of 500 mGy. To verify this hypothesis, we will investigate the regulation between DNA damage and mitochondrial dysfunction after HTO exposure in our future research.

In addition, low-dose-rate tritium β-ray radiation introduces a time aspect that increases the complexity of interpreting the different outcomes measured between doses of 500 mGy and 1 Gy. During long-term exposure, the DNA damage response is activated to respond to the damage, inducing a series of subsequent cell signal transduction. Studies have reported that the DNA damage response can be triggered at low doses (down to 20 mGy) [35]. Therefore, during tritium β-ray exposure, due to the long exposure time, DNA damage repair and subsequent activated cellular signaling can occur even if the irradiation from tritium β-rays is not finished. Moreover, accumulating evidence demonstrates that mitochondria play a vital role in the adaptive response through the mediation of mitochondrial metabolism [36]. Furthermore, studies have shown that the repair process of DNA double-strand breaks (DSBs) monitored by chromatin protein foci is slower following low-dose exposure [37,38]. ATP production is essential for the repair process of radiation damage, and inhibition of ATP production contributes to higher radiation sensitivity [20,39]. It is reasonable that a persistently high ATP level is observed after HTO exposure at a dose of 500 mGy, which differs from the results of ATP levels detected after X-ray irradiation. According to the high ATP level appearing from 6 h to 18 h post-HTO exposure at a dose of 500 mGy, it indicates that a sufficient ATP level can promote DNA repair processes, inducing subsequent activation events and determining the cell’s fate, whether it dies through apoptosis or survives. Consequently, increased apoptosis is observed from 6 h until 72 h post-HTO exposure, which is also the case for a dose of 1 Gy. For tritium β-ray radiation, the ATP and ROS levels at a dose of 1 Gy are lower than those at 500 mGy but higher than the sham-irradiated group within the first 12 h post-irradiation. This may be due to the fact that most DNA repair has occurred because the exposure time for 1 Gy is twice that of 500 mGy. Therefore, a decrease in ATP level from 12 h until 24 h post-HTO exposure at a dose of 1 Gy, accompanied by lower cell viability, is observed.

## 4. Conclusions

The radiation effects of normal B-lymphoblastoid cells after exposure to low-dose-rate irradiation from tritium β-rays have been investigated at doses of 500 mGy and 1 Gy. The results indicate that although there is little difference detected in cell viability at the low dose of 500 mGy between tritium β-rays and X-rays, the time course of post-irradiation ATP levels exhibited a dramatic difference between the tritium β-ray and X-ray irradiated groups. This difference was paralleled by the time course of the results of ROS levels measured in these same irradiation groups. These changes in mitochondrial function resulted in a continuous increase in apoptosis post-irradiation, following the low dose of 500 mGy. This suggests that mitochondrial function has the potential to be a new target for exploring the mechanisms related to the different effects of both low- and high-dose tritium β-ray irradiation.

## Data Availability

No new data were created.
